# *De novo* transcriptomes of 14 gammarid individuals for proteogenomic analysis of seven taxonomic groups

**DOI:** 10.1038/s41597-019-0192-5

**Published:** 2019-09-27

**Authors:** Yannick Cogne, Davide Degli-Esposti, Olivier Pible, Duarte Gouveia, Adeline François, Olivier Bouchez, Camille Eché, Alex Ford, Olivier Geffard, Jean Armengaud, Arnaud Chaumot, Christine Almunia

**Affiliations:** 1 Laboratoire Innovations technologiques pour la Détection et le Diagnostic (Li2D), Service de Pharmacologie et Immunoanalyse (SPI), CEA, INRA, F-30207 Bagnols-sur-Cèze, France; 2 Irstea, UR MALY Laboratoire d’écotoxicologie, centre de Lyon-Villeurbanne, F-69625 Villeurbanne, France; 3 GeT-PlaGe, Genotoul, INRA Auzeville, F-31320 Castanet-Tolosan, France; 4 School of Biological Sciences, Institute of Marine Sciences Laboratories, P04 9LY Portsmouth, United Kingdom

**Keywords:** Molecular ecology, Transcriptomics

## Abstract

Gammarids are amphipods found worldwide distributed in fresh and marine waters. They play an important role in aquatic ecosystems and are well established sentinel species in ecotoxicology. In this study, we sequenced the transcriptomes of a male individual and a female individual for seven different taxonomic groups belonging to the two genera *Gammarus* and *Echinogammarus*: *Gammarus fossarum A*, *G*. *fossarum B*, *G*. *fossarum C*, *Gammarus wautieri*, *Gammarus pulex*, *Echinogammarus berilloni*, and *Echinogammarus marinus*. These taxa were chosen to explore the molecular diversity of transcribed genes of genotyped individuals from these groups. Transcriptomes were *de novo* assembled and annotated. High-quality assembly was confirmed by BUSCO comparison against the Arthropod dataset. The 14 RNA-Seq-derived protein sequence databases proposed here will be a significant resource for proteogenomics studies of these ecotoxicologically relevant non-model organisms. These transcriptomes represent reliable reference sequences for whole-transcriptome and proteome studies on other gammarids, for primer design to clone specific genes or monitor their specific expression, and for analyses of molecular differences between gammarid species.

## Abstract (summary)

**Table:** 

Measurement(s)	transcription profiling assay
Technology Type(s)	RNA sequencing
Factor Type(s)	sex • species
Sample Characteristic - Organism	Gammarus • Echinogammarus
Sample Characteristic - Environment	habitat

Machine-accessible metadata file describing the reported data: 10.6084/m9.figshare.9777905

## Background & Summary

Gammarid amphipods are animals that typically measure a few millimetres long and present in a wide range of aquatic habitats^1^. In freshwater ecosystems, they are often the most dominant macro-invertebrates, representing a significant proportion of the total biomass, and they also play a central role within food webs. Indeed, they are a prey for many species, but are also predators for many invertebrate species. Amphipods are also scavengers and shredders, and detritivores involved in leaf litter breakdown, playing a central role in the decomposition of organic matter in general. Thus, they modulate the composition of freshwater communities of invertebrates^2^. Thanks to these essential roles, they have been the subject of many recent studies investigating their sensitivity to pollutants^3–7^.

Marine and freshwater resources are of the utmost importance for Life. Human-made chemical contaminants released into aquatic environments compromise the quality of water bodies, threatening the resident biodiversity, and the utility of such ecosystems. The quality of these environments should be evaluated not only by measuring the concentrations of pollutants present, but also by monitoring how Life is affected by the bioavailable pollutants present and their synergistic/antagonist effects^8^. To do this, biomonitoring with caged representative sentinel species has proved to be a valuable tool for efficient ecotoxicological studies^9–13^. Specific traits such as moult delay, growth impairment, or reproductive defects can be monitored on sensitive animals exposed to toxic environments. These data can be then integrated into a quantitative water quality index that can be used by stakeholders in charge of the aquatic ecosystem and water resource management^14^. Because of their abundance and central ecological roles, invertebrates are commonly employed as test organisms in marine and ecotoxicological assessments. Specifically, gammarids have been successfully used as sentinel species for freshwater ecosystems following investigations of their physiological responses to toxicants^15–23^ and biomonitoring in caging systems^9,12^. Specific biomarkers have been proposed and can be monitored by innovative methods such as tandem mass spectrometry^19,24–26^. Next-generation proteomics contributed to improving our knowledge of the molecular responses of gammarids to toxicants, and led to the proposal of a broad panel of appropriate biomarkers^27–30^. This approach was successful after developing a protein sequence database from an RNA-Seq transcriptome translated in all the possible reading frames. This proteogenomics concept was used to establish an extensive catalogue of protein sequences comprising 1873 mass-spectrometry-certified proteins, thus representing a significant amphipod proteomic resource^29^.

Despite this progress, molecular resources relating to gammarids remain scarce^31^. No gammarid whole genome sequence was available until very recently, when a first-draft genome of *Gammarus lacustris* was released comprising 443,304 scaffolds^32^. The genomes of two related amphipods, *Parhyale hawaiensis*^33^ and *Hyalella azteca*^34^, have also been sequenced. RNA-Seq datasets are now available for *P*. *hawaiensis*^35–37^, *Echinogammarus marinus*^38^, *Eogammarus possjeticus*^39^, *Gammarus fossarum*^29^, *Gammarus chevreuxi*^40^, *Gammarus pulex*^41^, and *Gammarus minus*^42^. However, these datasets are not of equal quality in terms of mRNA sequence coverage, which is a crucial parameter for proteogenomics interpretation^43^. They are assembled from mRNAs extracted from a pool of several animals or from specific tissues, and in some cases are no longer accessible as it is the case for *E*. *marinus* because the repository used no longer exists^44^.

The data presented in this article consist of assembled transcriptome sequences for 14 different gammarids, seven males and seven females, namely *Gammarus fossarum A* (*Müller type A*), *G*. *fossarum B* (*Müller type B*), *G*. *fossarum C* (*Müller type C*), *Gammarus wautieri*, *Gammarus pulex*, *Echinogammarus berilloni*, and *Echinogammarus marinus*. These transcriptomes were assembled and translated using the same pipelines (full length whole-organism mRNAs), and thus are of equivalent sequencing depth and quality across the different taxa studied. Starting material was extracted from single animals to avoid sequence heterogeneity. The transcriptomes have been annotated to serve as reference protein sequence databases for proteogenomics studies involving these sentinel animals that will be soon conducted to gain more basic knowledge and thus improve how aquatic environmental risks are assessed. For these future studies, an interesting strategy could be to interpret MS/MS shotgun data first on the most appropriate specific single-organism database, and then perform a follow-up search on a multi-organism database. The transcriptomes presented here will also serve in comparative analyses to better define the molecular diversity amongst gammarids and will be a valuable sequence resource for future ecotoxicological studies.

## Methods

### Experimental design

Freshwater gammarids were collected in four geographically-distant French rivers (Table 1). One population of *Gammarus fossarum* was sampled in north-eastern France (Seebach river), which was previously shown to harbour the cryptic type A subspecies according to the three types defined in Müller *et al*.^45^, Westram *et al*.^46^, and Weiss *et al*.^47^. The second river (Pollon River) situated in the mid-eastern area of France, corresponding to a sympatric situation, supplied organisms belonging to *Gammarus fossarum* type B, type C, and *Gammarus pulex* species. *Gammarus wautieri* were collected in the Galaveyson river in the Dauphiné region, and *Echinogammarus berilloni* organisms from a fourth river in south-western France (Saucats river). These freshwater gammarids were all collected using a hand net following kick-sampling, and subsequently transported to the laboratory. After maintaining them for 1 week in the laboratory – at 12 °C with a constant aeration, under a 16/8-h light/dark photoperiod in buckets containing water sampled from their respective rivers of origin, and with conditioned alder leaves as food source – couples in amplexus were isolated for species determination before RNA extraction. Pairs where the females had well-developed ovaries were selected. Embryos were removed from the marsupial pouch of females for five of these couples. Based on the description of the reproductive cycle in *Gammarus fossarum*^48^, for RNA extraction, we were able to select one couple per species in the last stage of the reproductive cycle (pre-moulting stage for the female) by retaining pairs where the females were carrying embryos at the end of their embryonic development stage (stage 4 or 5). For the marine species, *E*. *marinus* were collected from beneath seaweed in the intertidal zone in Portsmouth, southern England. These species correspond to the same population as used in a previous study^38^. After maintaining them for 1 month in the laboratory – at 10 °C under a 12 h light/12 h dark photoperiod in buckets with filtered natural seawater and fed with fucoid seaweed - organisms were transported live in damp seaweed from United Kingdom to France (one-day travel). They were subsequently maintained for a few hours in aquaria containing reconstituted seawater (salinity 30‰) before organism selection. For this species, it was not possible to recover couples in amplexus. One free-swimming male and one free-swimming female were isolated from the batch of organisms available. Stage 1 embryos were recovered from the female marsupium, indicating that this female was in a post-moulting stage.

**Table 1:** Sampling information and number of reads for each sample before and after filtering by mean quality for the 14 transcriptomes.

Species	Code Name	Sex	River	City	Country	GPS		Number of raw reads	Number of reads after filtering
*Echinogamarus berilloni*	EGSF	Female	Saucats	Saucats	France	44°39′34″N	0°34′25″W	80 482 966	80 277 434
*Echinogamarus berilloni*	EGSM	Male	Saucats	Saucats	France	44°39′34″N	0°34′25″W	90 372 154	90 118 242
*Echinogammarus marinus*	EGUF	Female	sea coast	Portsmouth	UK	50°47′41″N	1°01′50″W	85 032 246	84 652 454
*Echinogammarus marinus*	EGUM	Male	sea coast	Portsmouth	UK	50°47′41″N	1°01′50″W	70 768 994	70 540 528
Gammarus fossarum A*	GFAF	Female	Seebach	Fellering	France	47°53′31″N	6°58′53″E	81 959 830	81 543 116
Gammarus fossarum A*	GFAM	Male	Seebach	Fellering	France	47°53′31″N	6°58′53″E	95 167 986	94 695 372
Gammarus fossarum B*	GFBF	Female	Pollon	Saint-Maurice-de-Rémens	France	45°57′21″N	5°15′44″E	96 361 300	96 093 396
Gammarus fossarum B*	GFBM	Male	Pollon	Saint-Maurice-de-Rémens	France	45°57′21″N	5°15′44″E	85 125 996	84 758 816
Gammarus fossarum C*	GFCF	Female	Pollon	Saint-Maurice-de-Rémens	France	45°57′21″N	5°15′44″E	78 459 708	77 977 148
Gammarus fossarum C*	GFCM	Male	Pollon	Saint-Maurice-de-Rémens	France	45°57′21″N	5°15′44″E	75 598 166	75 407 534
*Gammarus pulex*	GPCF	Female	Pollon	Saint-Maurice-de-Rémens	France	45°57′21″N	5°15′44″E	84 202 086	83 965 920
*Gammarus pulex*	GPCM	Male	Pollon	Saint-Maurice-de-Rémens	France	45°57′21″N	5°15′44″E	89 235 492	89 025 410
*Gammarus wautieri*	GWF	Female	Galaveyson	Le Grand Serre	France	45°16′27″N	5°07′08″E	80 192 262	79 695 588
*Gammarus wautieri*	GWM	Male	Galaveyson	Le Grand Serre	France	45°16′27″N	5°07′08″E	63 959 618	63 638 482

*Müller type.

Species were first determined based on morphological criteria^49^. To distinguish between the three cryptic lineages, A, B, C, within the *G*. *fossarum* species, a molecular species assignment was carried out by amplifying the 5’ part of the mtDNA cytochrome c oxidase subunit I (COI) using universal primers (LCO1490 [GGT CAA ATC ATA AAG ATA TTG G] and HCO2198 [TAA ACT TCA GGG TGA CCA AAA AAT CA])^50^. Briefly, DNA was extracted from one or two pereopods (depending on individual size) cut from organisms before conditioning for RNA extraction. DNA was extracted using the Nucleospin tissue XS kit (Macherey-Nagel), and 10 ng of DNA for each organism was amplified. The PCR conditions consisted in 45 cycles of denaturation at 95 °C for 30 sec, annealing at 50 °C for 30 sec, and elongation at 72 °C for 1 min. PCR products were purified by ultrafiltration using the Nucleofast kit (Macherey-Nagel). Purified amplicons were prepared for sequencing using the BigDye Terminator v3.1 kit (ThermoFisher), and then sequenced on a DNA analyser ABI 3730XL (ThermoFisher). Sequencing data were analysed using the Sequencher 5.4.6 program (Genecodes). COI sequences (freely available from figshare, YC02_COI sequences and phylogenetic tree^51^) were aligned to build a phylogenetic tree including reference sequences from Weiss *et al*.^47^ and Lagrue *et al*.^52^. Using this phylogenetic tree (freely available from figshare, YC02_COI sequences and phylogenetic tree^51^) it is possible to position the COI sequences of the *Gammarus* organisms selected for RNA sequencing in relation to the published reference sequences (SeaView software^53^; BioNJ method based on J-C distance). The robustness of the different groupings was evaluated by a bootstrapping procedure (100 iterations). COI sequences were obtained for all *Gammarus* individuals, except for the female *G*. *fossarum C* as this individual was in precopulatory amplexus with the male COI-genotyped as *G*. *fossarum C*. However, in the same location (Pollon River), we also obtained the COI genotypes for 15 additional pairs, all of which were found to be non-heterospecific (4 *G*. *fossarum* B, 3 *G*. *fossarum* C, 8 *G*. *pulex*). Westraam *et al*.^46^ reported similar findings in the Glovelier river which harbours *G*. *fossarum* A and B, with only one heterospecific pair for a total of 64 genotyped pairs. Lagrue *et al*.^52^ also observed that mixed pairs are rare in the field for *Gammarus* lineages with a COI distance greater than 4%. Considering that the divergence between the COI-genotyped *G*. *fossarum* B and C specimens is about 17% in the Pollon River, it is very unlikely that this female does not belong to the *G*. *fossarum* C species.

### Dataset generation

Gammarids were placed in RNAlater (Sigma) and stored at 4 °C overnight. The RNAlater was then removed, and the organisms were snap frozen in liquid nitrogen and stored at −80 °C until RNA was extracted. Organisms were first homogenized in lysis buffer using a bead homogenizer and then RNAs were extracted using the Qiagen fibrous tissue kit (Qiagen). RNA quantity, quality and integrity were assessed by Nanodrop (Thermo Fisher) and Bioanalyzer (Agilent) analysis. RNA-Seq libraries were generated using the TruSeq stranded mRNA Sample Prep kit (Illumina). mRNA was purified using poly-(T) beads from 2 µg of each total RNA sample, then cleaved in segments of 155 bp on average (120–210 bp range). Subsequently, cleaved RNA fragments were primed with random hexamers and reverse-transcribed into first-strand cDNA. A second strand of cDNA was consecutively synthetized, and double-stranded cDNA was purified on beads. The 3′ ends of the blunt fragments obtained were then adenylated. Indexed adapters were ligated to the PCR-enriched cDNA fragments (11 cycles). Libraries were purified and quality-assessed using a Fragment Analyzer (Advanced Analytical Technologies). The 16 libraries were quantified by qPCR using the Kapa Library Quantification Kit (Roche). Their concentrations were normalized, multiplexed in a single pool. Libraries were then sequenced on two lanes of Hiseq3000 (Illumina) using a paired-end read length of 2 × 150 bp with the HiSeq Reagent Kits (Illumina). The two HiSeq lanes produced an average of 40.0 ± 8 million read pairs per library. Quality control of reads was performed by FastQC version V0.11.2 (Babraham Bioinformatics). Detailed results are freely available from figshare (YC02_QC data^51^). The data records are stored in 14 folders, each containing four folders per transcriptome.

### *De novo* assembly

For each sample, the forward or reverse reads were merged from two separate lanes. Data were filtered based on the mean Qphred score, with a threshold set at 16.99, and any remaining unpaired reads were removed using a homemade script. The numbers of reads for each sample before and after this filtering step are presented in Table 1. Trinity v2.4^54^ was used to assemble reads for each sample considering pair-end and strand orientation (-SS_lib type RF); all other Trinity parameters were set to their default values, with k set to 25, and minimum contig length to 200 bp.

### Assessing assembly quality

Transcriptome quality was assessed using Transrate v1.0.1^55^, which generates standard metrics and remapping statistics. No reference protein sequences were used for the assessment with Transrate. The main metrics are shown in Table 2. To validate the quality of all the assemblies, BUSCO v2.0^56^ was used. The database used for BUSCO analyses was Arthropoda_odb9 which contains 1066 orthologous genes at the nearest taxon level (*i*.*e*., Arthropods) available for *Gammarus*.

**Table 2:** Assembly quality metrics.

	EGSF	EGSM	EGUF	EGUM	GFAF	GFAM	GFBF	GFBM	GFCF	GFCM	GPCF	GPCM	GWF	GWM
n_seqs	166,100	211,358	162,914	133,658	182,439	383,876	325,379	344,409	280,883	324,661	245,224	257,575	214,232	183,988
largest	21,406	28,082	25,426	29,815	11,828	22,574	26,858	21,757	29,633	25,029	17,350	17,019	27,829	22,483
n_bases	178,852,651	228,738,512	168,030,154	142,457,935	118,459,292	283,956,781	259,691,927	263,406,154	226,877,323	236,552,608	198,832,295	180,448,306	186,939,687	144,019,426
mean_len	1076.8	1082.2	1031.4	1065.8	649.3	739.7	798.1	764.8	807.7	728.6	810.8	700.6	872.6	782.8
n_over_1k	42,496	54,408	44,211	38,307	31,373	76,176	66,143	67,014	57,497	58,066	50,528	44,353	49,495	38,349
n_over_10k	498	827	348	324	5	156	345	308	303	202	232	29	311	101
n_with_orf	35,470	58,284	38,503	30,621	32,784	78,940	62,829	65,479	53,123	56,151	40,810	33,985	46,313	40,639
mean_orf (%)	41.0	47.9	46.0	43.3	51.9	54.2	50.5	50.7	49.3	50.2	45.3	44.1	50.2	51.2
n90	355	361	340	357	270	282	285	284	289	273	283	271	310	300
n50	2646	2594	2278	2299	963	1240	1518	1354	1555	1290	1622	1187	1703	1328
n10	7494	7850	6812	6736	2978	4256	5442	5071	5593	4958	5522	4319	5767	4539
gc(%)	42.7	42.5	43.6	43.4	42.6	41.8	43.6	43.0	43.8	43.4	43.5	42.4	43.3	43.1
RMBT(%)*	91.7	94.4	89.6	91.9	88.2	83.9	90.1	82.7	87.7	86.1	81.9	84.8	87.2	86.5
G-RMBT(%)*	80.7	86.8	75.2	73.5	76.5	65.1	82.0	61.9	75.8	70.0	63.9	66.4	75.4	70.6
Score^#^	0.16	0.16	0.11	0.11	0.18	0.12	0.13	0.10	0.12	0.11	0.10	0.12	0.14	0.15

*RMBT means Reads Mapping Back on the Transcriptome; G-RMBT means Good Reads Mapping Back on the Transcriptome.

^#^Score calculated by Transrate.

### Annotation

For each sample, the transcripts were annotated using the Trinotate v3.1.1 annotation pipeline^54^. The Swissprot database was used as the main database, and amphipod proteins referenced on Uniref were used as a custom database. Similarity searches were performed with Blastx and Blastp, with an e-value cutoff set at 1e-2. Results from these searches were then used to generate the annotation report with the same e-value cutoff.

## Data Records

### Reads

Read sequences for each sample were deposited in the NCBI Sequence Reads Archive under accession Numbers SRR8089720^57^, SRR8089722–SRR8089725^58–61^, and SRR8089727–SRR8089735^62–70^, as indicated in Table 3 alongside the corresponding Bioproject and Biosample codes. The FastQC results for the 14 samples are freely available from figshare (YC02 _QC data)^51^. The data records are stored as 14 folders, each of which contain four folders per transcriptome.

**Table 3:** Accessions for the 14 transcriptomes.

Code Name	Transcriptome accession	Read accession	BioProject	BioSample
EGSF	GHCT01000000	*SRR8089732*	PRJNA497972	SAMN10259946
EGSM	GHCU01000000	*SRR8089733*	PRJNA497972	SAMN10259947
EGUF	GHCW01000000	*SRR8089734*	PRJNA497972	SAMN10259948
EGUM	GHCV01000000	*SRR8089735*	PRJNA497972	SAMN10259949
GFAF	GHCX01000000	*SRR8089727*	PRJNA497972	SAMN10259934
GFAM	GHCY01000000	*SRR8089728*	PRJNA497972	SAMN10259935
GFBF	GHCZ01000000	*SRR8089729*	PRJNA497972	SAMN10259936
GFBM	GHDA01000000	*SRR8089722*	PRJNA497972	SAMN10259937
GFCF	GHDC01000000	*SRR8089723*	PRJNA497972	SAMN10259938
GFCM	GHDB01000000	*SRR8089724*	PRJNA497972	SAMN10259939
GPCF	GHCP01000000	*SRR8089725*	PRJNA497972	SAMN10259940
GPCM	GHCQ01000000	*SRR8089720*	PRJNA497972	SAMN10259941
GWF	GHCR01000000	*SRR8089730*	PRJNA497972	SAMN10259944
GWM	GHCN01000000	*SRR8089731*	PRJNA497972	SAMN10259945

### Transcriptomes

Transcriptome assemblies were deposited in the NCBI Transcriptome Shotgun Assembly Sequence Database. These data have been deposited in GenBank under identifiers GHCN01000000^71^, GHCP01000000-GHCR01000000^72–74^, GHCT01000000-GHCZ01000000^75–81^, GHDA01000000-GHDC01000000^82–84^, as indicated in Table 3 alongside the corresponding Bioproject and Biosample codes.

### Proteogenomics databases

Translations of coding sequence regions were produced for each transcriptome from stop to stop codons by Transdecoder v3.0.1^54^, analysing only the top strand. The 500 longest ORFs were used for training, retaining 600-bp ORFs and only proteins with a minimum length of 50 amino acids. The 14 translations are freely available for download as FASTA files from figshare (YC02_Transcriptome translated ORFs^51^).

### Annotation

Annotations of each assembly are freely available for download as Excel files from figshare (YC02_Transcript annotations^51^). The folder contains 14 Excel files.

## Technical Validation

### Transrate

Transrate analyses showed good remapping of results, with more than 80% of reads remapped and most assemblies with more than 70% were classed as well mapped. Raw results from Transrate are freely available through figshare (YC02_ Transrate results)^51^.

### BUSCO

**Fig. 1:**
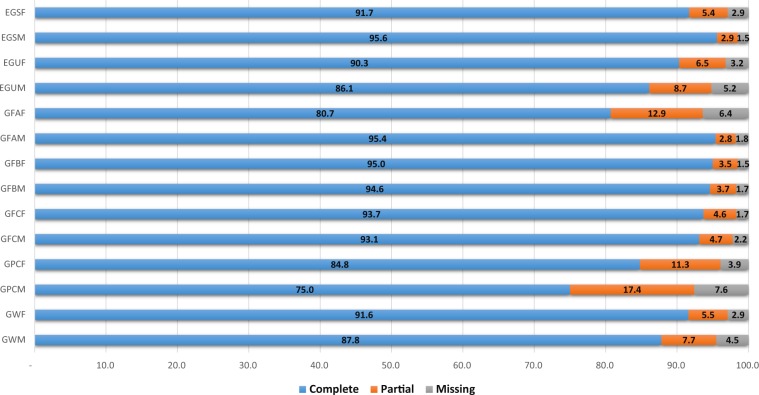
BUSCO assessment results for the 14 assembled transcriptomes.

A high level of single-copy ortholog retrieval was noted for the 14 assemblies, with at least a 75% ratio, as shown in Fig. 1. Furthermore, fewer than 8% of orthologs were missing in the worst case, and fewer than 5% were missing in 11 transcriptomes.

## Data Availability

Filtering before assembly was performed with an in-house Pythonv2.7 script, which is freely available (https://github.com/YannickCogne/Qfiltering). The script was automated with a bash script for each sample.

## References

[1] MacNeil C, Dick JTA, Elwood RW (1997). The trophic ecology of freshwater Gammarus Spp. (crustacea:amphipoda): problems and perspectives concerning the functional feeding group concept. Biological Reviews.

[2] Kelly DW, Dick JTA, Montgomery WI (2002). The functional role of Gammarus (Crustacea, Amphipoda): shredders, predators, or both?. Hydrobiologia.

[3] Arce-Funck J (2018). High stoichiometric food quality increases moulting organism vulnerability to pollutant impacts: An experimental test with Gammarus fossarum (Crustacea: Amphipoda). Sci Total Environ.

[4] Ganser B (2019). Wastewater alters feeding rate but not vitellogenin level of Gammarus fossarum (Amphipoda). Sci Total Environ.

[5] Könemann Sarah, Müller Yvonne, Tschentscher Daniel, Krauss Martin, Inostroza Pedro, Brückner Ira, Pinnekamp Johannes, Schiwy Sabrina, Hollert Henner (2019). Combination of In Situ Feeding Rate Experiments and Chemical Body Burden Analysis to Assess the Influence of Micropollutants in Wastewater on Gammarus pulex. International Journal of Environmental Research and Public Health.

[6] Munz NA, Fu Q, Stamm C, Hollender J (2018). Internal Concentrations in Gammarids Reveal Increased Risk of Organic Micropollutants in Wastewater-Impacted Streams. Environ Sci Technol.

[7] von Fumetti S, Blaurock K (2018). Effects of the herbicide Roundup(R) on the metabolic activity of Gammarus fossarum Koch, 1836 (Crustacea; Amphipoda). Ecotoxicology.

[8] Gouveia D (2018). Ecotoxicoproteomics: A decade of progress in our understanding of anthropogenic impact on the environment. J Proteomics.

[9] Besse JP (2013). Caged Gammarus fossarum (Crustacea) as a robust tool for the characterization of bioavailable contamination levels in continental waters: towards the determination of threshold values. Water Res.

[10] Chaumot, A., Geffard, O., Armengaud, J. & Maltby, L. In *Aquatic Ecotoxicology - Advancing tools for dealing with emerging risks* (eds Amiard-Triquet, C., Amiard, J.-C. & Mouneyrac, C.) 253–280 (Academic Press, London, 2015).

[11] Ciliberti A (2017). Caged Gammarus as biomonitors identifying thresholds of toxic metal bioavailability that affect gammarid densities at the French national scale. Water Res.

[12] Lacaze E (2011). DNA damage in caged Gammarus fossarum amphipods: a tool for freshwater genotoxicity assessment. Environ Pollut.

[13] Trapp J, Armengaud J, Salvador A, Chaumot A, Geffard O (2014). Next-generation proteomics: toward customized biomarkers for environmental biomonitoring. Environ Sci Technol.

[14] Coulaud R (2011). *In situ* feeding assay with Gammarus fossarum (Crustacea): Modelling the influence of confounding factors to improve water quality biomonitoring. Water Res.

[15] Barros S (2017). Chronic effects of triclocarban in the amphipod Gammarus locusta: Behavioural and biochemical impairment. Ecotoxicol Environ Saf.

[16] Chaumot A, Gos P, Garric J, Geffard O (2009). Additive vs non-additive genetic components in lethal cadmium tolerance of Gammarus (Crustacea): novel light on the assessment of the potential for adaptation to contamination. Aquat Toxicol.

[17] Correia AD, Lima G, Costa MH, Livingstone DR (2002). Studies on biomarkers of copper exposure and toxicity in the marine amphipod Gammarus locusta (Crustacea): I. Induction of metallothionein and lipid peroxidation. Biomarkers.

[18] Felten V (2008). Physiological and behavioural responses of Gammarus pulex (Crustacea: Amphipoda) exposed to cadmium. Aquat Toxicol.

[19] Jubeaux G (2012). Vitellogenin-like proteins in the freshwater amphipod Gammarus fossarum (Koch, 1835): functional characterization throughout reproductive process, potential for use as an indicator of oocyte quality and endocrine disruption biomarker in males. Aquat Toxicol.

[20] Kohler SA, Parker MO, Ford AT (2018). Species-specific behaviours in amphipods highlight the need for understanding baseline behaviours in ecotoxicology. Aquat Toxicol.

[21] Maltby L, Crane M (1994). Responses of Gammarus pulex (Amphipoda, Crustacea) to metalliferous effluents: identification of toxic components and the importance of interpopulation variation. Environ Pollut.

[22] Xuereb B, Chaumot A, Mons R, Garric J, Geffard O (2009). Acetylcholinesterase activity in Gammarus fossarum (Crustacea Amphipoda) Intrinsic variability, reference levels, and a reliable tool for field surveys. Aquat Toxicol.

[23] Xuereb B, Noury P, Felten V, Garric J, Geffard O (2007). Cholinesterase activity in Gammarus pulex (Crustacea Amphipoda): characterization and effects of chlorpyrifos. Toxicology.

[24] Gouveia D (2017). Ecotoxico-Proteomics for Aquatic Environmental Monitoring: First *in Situ* Application of a New Proteomics-Based Multibiomarker Assay Using Caged Amphipods. Environ Sci Technol.

[25] Gouveia D (2017). Assessing the relevance of a multiplexed methodology for proteomic biomarker measurement in the invertebrate species Gammarus fossarum: A physiological and ecotoxicological study. Aquat Toxicol.

[26] Simon R (2010). Mass spectrometry assay as an alternative to the enzyme-linked immunosorbent assay test for biomarker quantitation in ecotoxicology: application to vitellogenin in Crustacea (Gammarus fossarum). J Chromatogr A.

[27] Trapp J (2016). High-throughput proteome dynamics for discovery of key proteins in sentinel species: Unsuspected vitellogenins diversity in the crustacean Gammarus fossarum. J Proteomics.

[28] Trapp J (2015). Proteomic investigation of male Gammarus fossarum, a freshwater crustacean, in response to endocrine disruptors. J Proteome Res.

[29] Trapp J (2014). Proteogenomics of Gammarus fossarum to document the reproductive system of amphipods. Mol Cell Proteomics.

[30] Trapp, J. *et al*. Digging Deeper Into the Pyriproxyfen-Response of the Amphipod Gammarus fossarum With a Next-Generation Ultra-High-Field Orbitrap Analyser: New Perspectives for Environmental Toxicoproteomics. *Frontiers in Environmental Science* **6**, 54 (2018).

[31] Armengaud J (2014). Non-model organisms, a species endangered by proteogenomics. J Proteomics.

[32] Jin S (2019). Identification of Candidate Genes for the Plateau Adaptation of a Tibetan Amphipod, Gammarus lacustris, Through Integration of Genome and Transcriptome Sequencing. Front Genet.

[33] Kao, D. *et al*. The genome of the crustacean Parhyale hawaiensis, a model for animal development, regeneration, immunity and lignocellulose digestion. *Elife* **5**, e20062 (2016). 10.7554/eLife.20062 PMC5111886 27849518

[34] Poynton HC (2018). The Toxicogenome of Hyalella azteca: A Model for Sediment Ecotoxicology and Evolutionary Toxicology. Environ Sci Technol.

[35] Blythe MJ (2012). High through-put sequencing of the Parhyale hawaiensis mRNAs and microRNAs to aid comparative developmental studies. PLoS One.

[36] Nestorov P, Battke F, Levesque MP, Gerberding M (2013). The maternal transcriptome of the crustacean Parhyale hawaiensis is inherited asymmetrically to invariant cell lineages of the ectoderm and mesoderm. PLoS One.

[37] Zeng V (2011). *De novo* assembly and characterization of a maternal and developmental transcriptome for the emerging model crustacean Parhyale hawaiensis. BMC Genomics.

[38] Short S (2014). Crustacean intersexuality is feminization without demasculinization: implications for environmental toxicology. Environ Sci Technol.

[39] Chen J, Liu H, Cai S, Zhang H (2019). Comparative transcriptome analysis of Eogammarus possjeticus at different hydrostatic pressure and temperature exposures. Sci Rep.

[40] Truebano M, Tills O, Spicer JI (2016). Embryonic transcriptome of the brackishwater amphipod Gammarus chevreuxi. Mar Genomics.

[41] Gismondi E, Thome JP (2016). Transcriptome of the freshwater amphipod Gammarus pulex hepatopancreas. Genom Data.

[42] Carlini DB, Fong DW (2017). The transcriptomes of cave and surface populations of Gammarus minus (Crustacea: Amphipoda) provide evidence for positive selection on cave downregulated transcripts. PLoS One.

[43] Trapp J (2016). Proteogenomic insights into the core-proteome of female reproductive tissues from crustacean amphipods. J Proteomics.

[44] Jones M, Blaxter M (2013). afterParty: turning raw transcriptomes into permanent resources. BMC Bioinformatics.

[45] Muller J (2000). Mitochondrial DNA variation and the evolutionary history of cryptic Gammarus fossarum types. Mol Phylogenet Evol.

[46] Westram AM, Jokela J, Baumgartner C, Keller I (2011). Spatial distribution of cryptic species diversity in european freshwater amphipods (Gammarus fossarum) as revealed by pyrosequencing. PLoS One.

[47] Weiss M, Macher JN, Seefeldt MA, Leese F (2014). Molecular evidence for further overlooked species within the Gammarus fossarum complex (Crustacea: Amphipoda). Hydrobiologia.

[48] Geffard O (2010). Ovarian cycle and embryonic development in Gammarus fossarum: application for reproductive toxicity assessment. Environ Toxicol Chem.

[49] Piscart, C. & Bollache, L. *Crustacés amphipodes de surface: gammares d’eau douce*. (Association Française de Limnologie, 2012).

[50] Folmer O, Black M, Hoeh W, Lutz R, Vrijenhoek R (1994). DNA primers for amplification of mitochondrial cytochrome c oxidase subunit I from diverse metazoan invertebrates. Mol Mar Biol Biotechnol.

[51] Cogne Y (2019). figshare.

[52] Lagrue C (2014). Confrontation of cryptic diversity and mate discrimination within Gammarus pulex and Gammarus fossarum species complexes. Freshwater Biology.

[53] Gouy M, Guindon S, Gascuel O (2010). SeaView version 4: A multiplatform graphical user interface for sequence alignment and phylogenetic tree building. Mol Biol Evol.

[54] Grabherr MG (2011). Full-length transcriptome assembly from RNA-Seq data without a reference genome. Nat Biotechnol.

[55] Smith-Unna R, Boursnell C, Patro R, Hibberd JM, Kelly S (2016). TransRate: reference-free quality assessment of *de novo* transcriptome assemblies. Genome Res.

[56] Seppey M, Manni M, Zdobnov EM (2019). BUSCO: Assessing Genome Assembly and Annotation Completeness. Methods Mol Biol.

[57] (2019). NCBI Sequence Read Archive.

[58] (2019). NCBI Sequence Read Archive.

[59] (2019). NCBI Sequence Read Archive.

[60] (2019). NCBI Sequence Read Archive.

[61] (2019). NCBI Sequence Read Archive.

[62] (2019). NCBI Sequence Read Archive.

[63] (2019). NCBI Sequence Read Archive.

[64] (2019). NCBI Sequence Read Archive.

[65] (2019). NCBI Sequence Read Archive.

[66] (2019). NCBI Sequence Read Archive.

[67] (2019). NCBI Sequence Read Archive.

[68] (2019). NCBI Sequence Read Archive.

[69] (2019). NCBI Sequence Read Archive.

[70] (2019). NCBI Sequence Read Archive.

[71] (2019). GenBank.

[72] (2019). GenBank.

[73] (2019). GenBank.

[74] (2019). GenBank.

[75] (2019). GenBank.

[76] (2019). GenBank.

[77] (2019). GenBank.

[78] (2019). GenBank.

[79] (2019). GenBank.

[80] (2019). GenBank.

[81] (2019). GenBank.

[82] (2019). GenBank.

[83] (2019). GenBank.

[84] (2019). GenBank.

